# Model Predictive Control-Based Assist-as-Needed Strategy for Reducing Motor Slacking in Robot-Assisted Rehabilitation

**DOI:** 10.3390/s26092740

**Published:** 2026-04-28

**Authors:** Choonggun Kim, Youngjin Moon, Jaesoon Choi

**Affiliations:** 1 Department of Mechanical Engineering, Sogang University, Seoul 04107, Republic of Korea; kcg1755@sogang.ac.kr; 2 Department of Biomedical Engineering, Asan Medical Center, University of Ulsan College of Medicine, Seoul 05505, Republic of Korea; jacobian@amc.seoul.kr

**Keywords:** Assist-as-Needed, model predictive control, upper-limb rehabilitation robot, motor slacking, human–robot interaction, admittance control

## Abstract

This study proposes a model predictive control (MPC)-based Assist-as-Needed (AAN) strategy for upper-limb rehabilitation robots, with particular emphasis on mitigating motor slacking. In conventional error-based AAN approaches, robotic assistance is regulated through a single coefficient tied to the tracking error; thus, a reduction in voluntary effort is absorbed into the assistive channel and remains obscured by a small tracking error. The proposed method decouples this mechanism by introducing a two-channel admittance structure, in which the robotic-assistance gain $A_{k}$ and the user-participation-reflection gain $B_{k}$ are jointly optimized within a single convex MPC formulation. The cost function addresses trajectory tracking, participation-aware force alignment, assistance suppression, and passivity, enforced through energy-tank constraints. The controller was validated in two experiments on a mobile upper-limb rehabilitation robot. The first experiment confirmed differential adaptation of $A_{k}$ and $B_{k}$ across three instructed contribution levels, with the participation ratio increasing from 0.103 to 0.879 as the contribution shifted from insufficient to appropriate. The second experiment compared the controller with an error-based AAN baseline and a forgetting-factor AAN baseline under an induced motor-slacking condition, in which the task-direction contribution was reduced to 45%. Under an identical synthesized input, the proposed controller yielded a lower aggregate human-contribution ratio of 0.282, compared with 0.595 and 0.535 for the two baselines, respectively. This indicates that the externally imposed reduction in participation was represented more explicitly in the controller allocation, rather than being masked by error-driven assistive compensation. These results suggest that the proposed approach extends AAN control toward a participation-preserving, anti-slacking strategy for robot-assisted rehabilitation.

## 1. Introduction

Neurological injuries, including stroke, traumatic brain injury, and spinal cord injury, substantially impair activities of daily living and reduce patient independence, highlighting the need for early, repetitive, task-oriented motor training [1,2]. Conventional manual rehabilitation is constrained by therapist dependency, physical demand, and inconsistent training intensity [3]. Robot-assisted rehabilitation addresses these limitations through superior repeatability, reproducibility, and the capacity for intensive, quantifiable practice [4]. Research has since advanced toward adaptive systems that tailor assistance to patient-specific performance while enabling quantitative assessment of participation and therapeutic outcomes [5,6].

Existing control strategies for upper-limb rehabilitation robots fall into two categories. The first, encompassing position-tracking, impedance, and admittance control, ensures stable execution of predefined reference trajectories [7,8], providing safe, predictable motion and a guaranteed level of task completion, especially for severely impaired patients. The second is the Assist-as-Needed (AAN) paradigm, which modulates robotic assistance according to the patient’s residual motor capacity and instantaneous task performance [9,10]. AAN preserves volitional control when the patient can move independently, providing assistance only when performance falls below an acceptable level, thereby promoting active engagement. Recent AAN architectures determine assistance from three information sources: task-performance errors, physiological or behavioral signals, and human–robot interaction forces [5,11,12].

Despite these developments, both paradigms remain structurally susceptible to motor slacking. Tracking-based control focuses on reproducing the desired motion irrespective of the patient’s active contribution and is therefore prone to inducing passive dependence on the robotic system [7,13]. Although AAN is intended to reduce unnecessary assistance, many existing designs continue to prioritize task completion and trajectory-error minimization [9]: larger tracking deviations elicit stronger restorative forces or higher assistive stiffness, effectively suppressing the observable error; yet ultimate responsibility for task completion continues to rest with the robot. Consequently, the structural tendency toward diminished voluntary participation is not resolved at the architectural level.

Recent studies have incorporated optimization-based formulations or participation-aware metrics into AAN design. On the MPC side, an optimal impedance MPC scheme has been proposed for robot-aided rehabilitation [14], and a Laguerre-based predictive controller has been applied to a wearable upper-limb rehabilitation robot [15]. On the participation-aware AAN side, an iterative framework adjusts assistance through an interaction factor across repeated trials [16]; unaffected-limb motion has been exploited to estimate the required assistance [12]; and real-time adaptive controllers have modulated assistance based on task-performance errors and multimodal user responses [5,6]. These studies have advanced optimization-based and participation-aware rehabilitation control, yet they share a common structural limitation: robotic assistance is regulated as a single channel coupled, directly or indirectly, to the tracking error or an externally estimated effort proxy. Consequently, a reduction in voluntary effort is absorbed into the assistive channel and manifests only as increased robotic assistance despite a small tracking error, rather than being represented as a distinct quantity at the controller level.

This study reconsiders motor slacking from a control-structural perspective. Rather than treating it merely as a consequence of excessive assistance, motor slacking is interpreted as an adaptive phenomenon in which the patient gradually learns, through repeated interaction, that the robot will ensure task completion and therefore reduces voluntary effort [13,17]. To operationalize this concept for controller design, voluntary effort is approximated by the task-direction projection of the user interaction force, $t_{k}^{⊤}F_{\mathrm{inter},k}$, where $t_{k}$ is the unit task-direction vector and $F_{\mathrm{inter},k}$ is the measured interaction force. Motor slacking is then operationally interpreted as a decrease in this quantity under otherwise matched task conditions; the precise control formulation is introduced in Section 3. The central issue is therefore not the magnitude of robotic assistance itself, but whether the control objective is structured such that task completion does not suppress the regulation of task-direction user contribution. Suppressing motor slacking requires an architecture that preserves tracking performance while limiting redundant assistance, a multi-objective problem naturally addressed by model predictive control (MPC) [14,15].

Motivated by this perspective, this paper proposes an MPC-based AAN control architecture for upper-limb rehabilitation robots that limits the controller-side compensation mechanism through which motor slacking can emerge. Rather than scheduling a single assistive channel, the proposed framework regulates the robotic-assistance gain $A_{k}$ and the user-participation-reflection gain $B_{k}$ as two independent decision variables within a single convex MPC formulation. The main contributions are as follows:

- Motor slacking is operationally formalized as a reduction in the task-direction projection of the voluntary interaction force, yielding a measurable control quantity on which the controller objectives are directly constructed [13,17].
- A two-channel convex MPC formulation is developed in which user participation is treated as an explicit decision variable. The two-channel admittance structure optimizes $A_{k}$ and $B_{k}$ independently within a single convex MPC that jointly addresses tracking, participation reflection, assist suppression, and passivity. This distinguishes the proposed design from prior single-channel MPC approaches [14,15] and from error- or factor-scheduled AAN approaches [5,12,16], in which participation is absorbed into the assistive channel.
- The proposed controller is experimentally validated at the controller level on a mobile upper-limb rehabilitation robot against two representative AAN baselines, namely an error-based AAN scheme and a forgetting-factor AAN scheme. The evaluation examines whether reduced task-direction human contribution becomes observable in the controller allocation rather than being masked by low tracking error.

The remainder of this paper is organized as follows. Section 2 presents the hardware and software architecture of the proposed rehabilitation robotic system. Section 3 describes the design methodology of the RMS-AAN algorithm that incorporates motor slacking into the control objective. Section 4 reports the experimental setup and performance evaluation results. Finally, Section 5 concludes the paper and discusses directions for future research.

## 2. System Design and Implementation

### Configuration of the System

**Figure 1:**
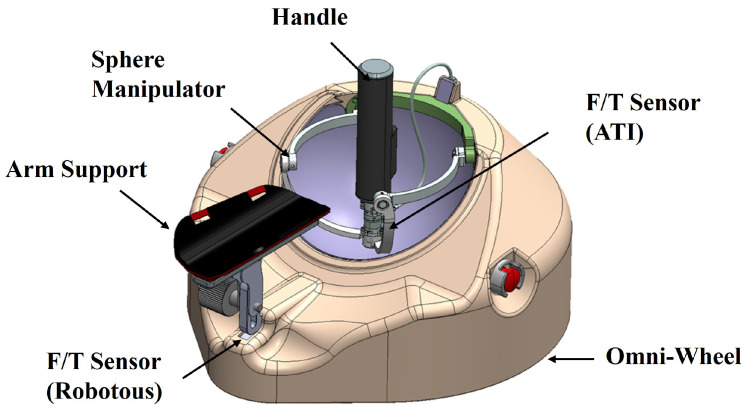
Configuration of the mobile upper-limb robot system.

As illustrated in Figure 1, the proposed rehabilitation robot adopts a mobile platform–spherical manipulator architecture to generate upper-limb training motions while maintaining safe physical interaction with the user. The platform produces planar translational motion together with yaw rotation, and the spherical manipulator provides wrist-orientation support and physical interfacing at the distal end. In the present study, the control formulation targets the planar rehabilitation task in the (x,y) space; orientation-related motion is treated as an auxiliary function rather than the primary focus of the proposed MPC design.

The mobile platform, equipped with three omni wheels, generates planar motion in the *x* and *y* directions at the hand task point while simultaneously regulating the platform’s yaw angle to adjust the gross orientation of the upper limb, thereby serving as the primary actuation unit for planar rehabilitation motion. The 2-DOF spherical manipulator supports wrist posture accommodation, selectively targets rotational components depending on the user’s gripping configuration, and stabilizes the wrist as a distal physical interface during training.

During training, the user’s wrist is secured to the spherical manipulator, and the forearm is supported on an arm support mounted on the mobile platform. Force/torque (F/T) sensors installed at the distal end of the spherical manipulator and at the arm support measure user–robot interaction at both contact locations; the measured wrenches are transformed into a common task-space frame and combined into the resultant interaction force $F_{\mathrm{inter}}$, which is applied to the admittance controller.

**Figure 2:**
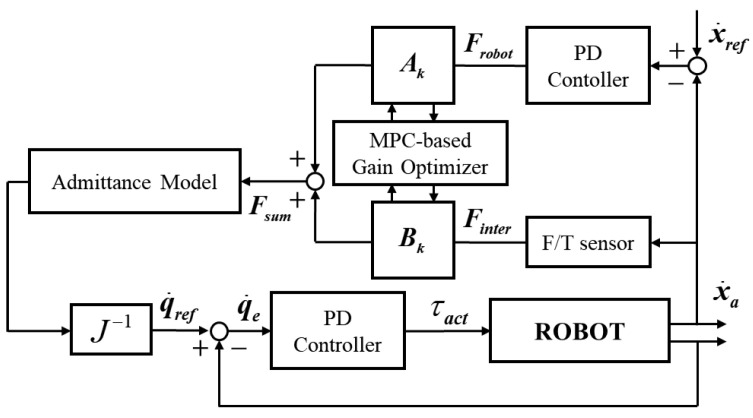
Control architecture of the proposed mobile upper-limb rehabilitation robot.

The measured interaction force drives the admittance-control loop, as shown in Figure 2, generating compliant task-space motion consistent with the user input. Although the system supports both planar translation and forearm-orientation motion, the RMS-AAN controller addresses only the planar task; the task variable is defined as $x_{a}={\left[x_{a}\;\;y_{a}\right]}^{⊤}$.

The admittance loop computes the target control position. The virtual admittance model is a second-order linear system with constant, diagonal, positive-definite mass and damping matrices, decoupling each planar axis into an independent admittance channel while preserving a physically interpretable compliant response to user interaction. The control law is written as

(1) $$M_{v}{\ddot{x}}_{a}+B_{v}{\dot{x}}_{a}=A_{c}\left(t\right)\;F_{\mathrm{robot}}+B_{c}\left(t\right)\;F_{\mathrm{inter}},\;0\leq A_{c}\left(t\right)\leq 1,\;0\leq B_{c}\left(t\right)\leq 1$$

where $M_{v}\in R^{2\times 2}$ and $B_{v}\in R^{2\times 2}$ denote the virtual mass and damping matrices, respectively. These matrices determine the desired compliance characteristics of the planar rehabilitation dynamics. The term $F_{\mathrm{robot}}$ denotes the robot-generated assistive force for reference tracking and is computed using a PD law:

(2) $$F_{\mathrm{robot}}=K_{p}\left(x_{\mathrm{ref}}-x_{a}\right)+K_{d}\left({\dot{x}}_{\mathrm{ref}}-{\dot{x}}_{a}\right)$$

The time-varying continuous-time gains $A_{c}\left(t\right)$ and $B_{c}\left(t\right)$ independently modulate the contributions of the robot-assistance channel and the user-participation channel, respectively. In particular, $A_{c}\left(t\right)=1$ and $B_{c}\left(t\right)=0$ correspond to a robot-dominant task-guarantee mode, whereas $A_{c}\left(t\right)=0$ and $B_{c}\left(t\right)=1$ correspond to a human-driven compliant mode. Intermediate values continuously interpolate between these two limiting cases, thereby allowing the controller to balance task completion and user participation.

For model derivation, the continuous-time planar dynamics corresponding to (1) are expressed as

(3) $${\ddot{x}}_{a}=M_{v}^{-1}\left(-B_{v}{\dot{x}}_{a}+A_{c}\left(t\right)\;F_{\mathrm{robot}}+B_{c}\left(t\right)\;F_{\mathrm{inter}}\right)$$

where $A_{c}\left(t\right)$ and $B_{c}\left(t\right)$ denote the instantaneous assistance and participation gains in continuous time. For MPC prediction, these gains are sampled at each control step and represented by $A_{k}$ and $B_{k}$, respectively. In addition, the predicted assistive force is treated as an exogenous quantity in the prediction model such that the state-update equation remains affine in the optimization variables. A detailed justification of this treatment is provided in Section 3.

Let the discrete-time state be defined as $z_{k}={\left[x_{a,k}^{⊤},\;{\dot{x}}_{a,k}^{⊤}\right]}^{⊤}\in R^{4}$ and $u_{k}={\left[A_{k},\;B_{k}\right]}^{⊤}\in R^{2}$. Using an explicit forward-Euler discretization with sampling period $T_{s}$, the prediction model is written as

(4) $$z_{k+1}=Az_{k}+G_{k}u_{k}$$

$$A=\left[\begin{array}{cc} I_{2} & T_{s}\;I_{2}\\ 0_{2\times 2} & I_{2}-T_{s}M_{v}^{-1}B_{v} \end{array}\right],\;G_{k}=\left[\begin{array}{cc} 0_{2\times 1} & 0_{2\times 1}\\ T_{s}M_{v}^{-1}{\hat{F}}_{\mathrm{robot},k} & T_{s}M_{v}^{-1}F_{\mathrm{inter},k} \end{array}\right]$$

Here, ${\hat{F}}_{\mathrm{robot},k}$ is obtained from (2) and treated as a known exogenous signal such that $G_{k}\in R^{4\times 2}$ preserves affine dependence on $u_{k}$. In (4), $A_{k}$ and $B_{k}$ independently scale the robot-assistance and user-participation channels, distinguishing the proposed two-channel admittance architecture from conventional single-coefficient mixing.

The task-space velocity command generated by the admittance layer is subsequently mapped to the actuator space through the inverse Jacobian, yielding the joint-space velocity reference ${\dot{\theta }}_{\mathrm{ref}}$. This reference is then tracked by a lower-level PID controller to produce the actuator torque command $\tau_{\mathrm{act}}$, thereby driving the robot to realize the desired rehabilitation motion.

## 3. Reduced Motor Slacking Assist-as-Needed Control

To address motor slacking in conventional AAN control, this paper proposes the Reduced Motor Slacking AAN (RMS-AAN) framework, which preserves active user participation while maintaining stable task execution. The controller is formulated as an MPC problem over a horizon *N*, jointly optimizing the assistance gain $A_{k}$ and the participation-reflection gain $B_{k}$.

The control design is organized around three objectives. The first objective is tracking performance, which ensures task execution accuracy and bounded transient error. The second objective is participation reflection, which encourages the controller to preserve task-direction human contribution when the user is actively assisting the motion. The third objective is assist suppression, which penalizes unnecessarily robot-dominant behavior and reduces abrupt gain variation. As these objectives are partially conflicting, they are balanced through a convex quadratic program (QP) with explicit gain and passivity constraints.

### 3.1. Modeling Assumptions and Prediction Model

The linear MPC formulation rests on four assumptions. First, $M_{v}$ and $B_{v}$ are constant, diagonal, and positive-definite, rendering the planar admittance dynamics linear time-invariant; Coriolis, centrifugal, and gravitational terms are neglected because the task is quasi-planar and quasi-horizontal. Second, the diagonal structure of $M_{v}$ and $B_{v}$ decouples the two Cartesian coordinates into independent single-DOF admittance channels, a standard approximation in planar end-effector admittance control [18]. Third, $F_{\mathrm{inter}}$ is the resultant task-space force reconstructed from two calibrated six-axis F/T sensors after bias removal, frame transformation, and first-order low-pass filtering. Fourth, (1) contains no explicit virtual stiffness term; restoration toward the reference trajectory is instead provided by the PD-based assistive force in (2), where $K_{p}$ acts as an effective task-space stiffness scaled by $A_{k}$.

The continuous-time dynamics (3) are discretized via explicit forward Euler to keep the prediction model affine in the MPC variables. The numerical stability of the velocity subdynamics $I_{2}-T_{s}M_{v}^{-1}B_{v}$ requires

(5) $$T_{s}<\frac{2}{\max_{i}\lambda_{i}\;\left(M_{v}^{-1}B_{v}\right)}$$

which is not an asymptotic Schur-stability condition for the full matrix A, since A retains position integrators by construction. With $M_{v}=2I_{2}$ and $B_{v}=40I_{2}$, (5) yields $T_{s}<0.1\;s$, which is satisfied by the selected $T_{s}=10\;\mathrm{ms}$. Interaction-port stability under time-varying $A_{k}$ and $B_{k}$ is addressed separately by the energy-tank constraints in Section 3.3.

A further issue is that ${\hat{F}}_{\mathrm{robot},k}$ depends on the predicted state via (2); direct substitution into the prediction model would yield a bilinear formulation. To preserve a convex QP, ${\hat{F}}_{\mathrm{robot},k+i|k}$ is instead precomputed from a nominal trajectory obtained by forward integrating the model with a warm-started input sequence shifted from the previous step. Both ${\hat{F}}_{\mathrm{robot},k+i|k}$ and $F_{\mathrm{inter},k+i|k}$ then enter the prediction model as known exogenous quantities, yielding an affine linear time-varying (LTV) structure. The approximation error is reduced in practice by the gain-rate penalties $J_{\Delta A}$ and $J_{\Delta B}$.

### 3.2. Design of the RMS-AAN Cost Function

The proposed cost function is designed such that tracking quality, participation reflection, and assist suppression are handled explicitly but within a single convex optimization problem.

Tracking performance is quantified by the position and velocity errors between the reference and predicted trajectories. An $\ell_{2}$-norm-based cost is adopted because it increases nonlinearly with error magnitude, thereby allowing aggressive correction in large-error regions while avoiding excessive sensitivity in small-error regions [14,15,19,20]:

(6) $$J_{track}=\sum_{i=0}^{N-1}\left(∥x_{a,k+i|k}-x_{ref,k+i|k}{∥}_{Q_{x}}^{2}+{∥{\dot{x}}_{a,k+i|k}-{\dot{x}}_{ref,k+i|k}∥}_{Q_{v}}^{2}\right)+{∥x_{a,k+N|k}-x_{ref,k+N|k}∥}_{Q_{f}}^{2}$$

where $Q_{x}⪰0$, $Q_{v}⪰0$, and $Q_{f}⪰0$ are the position-, velocity-, and terminal-error weight matrices, respectively. Although zero entries are permissible in principle, all three are chosen as strictly positive diagonal matrices to preserve QP convexity, maintain numerical conditioning, and retain tracking regularization along both task-space axes.

Let $h_{k+i|k}=t_{k+i}^{⊤}F_{\mathrm{inter},k+i|k}$ denote the task-direction projection of the user interaction force, where $t_{k+i}$ is the unit task-progress direction. Motor slacking is operationally associated with a reduction in $h_{k+i|k}$ under matched task conditions. To preserve this task-direction human contribution at the controller level, an alignment cost is introduced:

(7) $$J_{align}=\sum_{i=0}^{N-1}S{\left(B_{k+i|k}\;t_{k+i}^{⊤}F_{\mathrm{inter},k+i|k}-t_{k+i}^{⊤}{\hat{F}}_{\mathrm{robot},k+i|k}\right)}^{2}$$

where S≥0 is a scalar alignment weight. This term encourages the participation-reflection gain $B_{k+i|k}$ to increase when the user generates force components aligned with the task-progress direction [9,13,16,21,22].

Only the task-direction projection is used in (7) because the control objective centers on progression along the reference path; orthogonal components are lateral deviations already penalized by (6). The asymmetry in (7), where $B_{k+i|k}$ multiplies $F_{\mathrm{inter},k+i|k}$ but not ${\hat{F}}_{\mathrm{robot},k+i|k}$, is intentional: the robot term is the task-achieving reference channel, while the human term is the channel whose reflected contribution is regulated.

Since $F_{\mathrm{inter}}$ may fluctuate over time, a zero-order hold approximation is applied over the short prediction horizon:

(8) $$F_{\mathrm{inter},k+i|k}\approx F_{\mathrm{inter},k},\;i=0,1,\ldots ,N-1$$

This approximation is acceptable because the QP is re-solved at every sampling step, and the horizon is short relative to the expected variation in voluntary upper-limb interaction forces. Nevertheless, abrupt within-horizon fluctuations in user force may reduce prediction accuracy, and this should be regarded as a limitation of the present linearized formulation.

To preserve convexity, ${\hat{F}}_{\mathrm{robot},k+i|k}$ is precomputed from a nominal admittance trajectory rather than being coupled directly to the predicted optimization state:

(9) $${\hat{F}}_{\mathrm{robot},k+i|k}=K_{p}\;\left(x_{ref,k+i|k}-{\hat{x}}_{a,k+i|k}\right)+K_{d}\;\left({\dot{x}}_{ref,k+i|k}-{\dot{\hat{x}}}_{a,k+i|k}\right)$$

where $\left({\hat{x}}_{a,k+i|k},{\dot{\hat{x}}}_{a,k+i|k}\right)$ is obtained by forward integrating the prediction model with the shifted previous input sequence.

The assist-suppression term penalizes the magnitude of the scaled robotic assistive force:

(10) $$J_{assist}=\sum_{i=0}^{N-1}{∥A_{k+i|k}{\hat{F}}_{\mathrm{robot},k+i|k}∥}_{R}^{2}$$

where R⪰0 is the assistive-force weighting matrix.

Assistance is further shaped through a desired assistance reference $A_{des,k+i|k}$ that depends on the predicted tracking-error magnitude. The scalar tracking errors are defined as

(11) $$e_{k+i|k}^{x}={∥x_{ref,k+i|k}-x_{a,k+i|k}∥}_{2},\;e_{k+i|k}^{\dot{x}}={∥{\dot{x}}_{ref,k+i|k}-{\dot{x}}_{a,k+i|k}∥}_{2}$$

and the desired assist gain is

(12) $$A_{des,k+i|k}={\mathrm{sat}}_{\left[0,1\right]}\;\left(K_{A,x}\;{\mathrm{sat}}_{\left[0,1\right]}\;\left(\frac{e_{k+i|k}^{x}}{e_{ref}^{x}}\right)+K_{A,\dot{x}}\;{\mathrm{sat}}_{\left[0,1\right]}\;\left(\frac{e_{k+i|k}^{\dot{x}}}{e_{ref}^{\dot{x}}}\right)\right)$$

where $e_{ref}^{x}$ and $e_{ref}^{\dot{x}}$ are the maximum tolerable position and velocity errors used for normalization. The inner saturations prevent a single error channel from dominating the assist demand; the outer saturation enforces compatibility with the gain bound $0\leq A_{k+i|k}\leq 1$. The gain $A_{k+i|k}$ is then shaped toward this reference by

(13) $$J_{A}=\sum_{i=0}^{N-1}\omega_{A}{\left(A_{k+i|k}-A_{des,k+i|k}\right)}^{2}$$

To suppress discontinuous intervention and high-frequency gain oscillations, smoothing penalties are imposed on both gain sequences. Let $A_{k-1}$ and $B_{k-1}$ denote the gains applied at the preceding control step; the smoothing penalties are defined as

(14) $$J_{\Delta A}=\omega_{\Delta A}{\left(A_{k|k}-A_{k-1}\right)}^{2}+\sum_{i=1}^{N-1}\omega_{\Delta A}{\left(A_{k+i|k}-A_{k+i-1|k}\right)}^{2}$$

(15) $$J_{\Delta B}=\omega_{\Delta B}{\left(B_{k|k}-B_{k-1}\right)}^{2}+\sum_{i=1}^{N-1}\omega_{\Delta B}{\left(B_{k+i|k}-B_{k+i-1|k}\right)}^{2}$$

This formulation explicitly ties the first predicted move to the previously applied gains, avoiding the indexing ambiguity that would otherwise arise at i=0.

The total objective, comprising $J_{track}$, $J_{align}$, $J_{assist}$, $J_{A}$, $J_{\Delta A}$, and $J_{\Delta B}$, is quadratic and convex in the decision variables, and is therefore compatible with real-time QP implementation.

The cost terms contain inner-loop weights $\left(Q_{x},Q_{v},Q_{f},S,R,\omega_{A}\right)$ and outer coefficients $\left(\lambda_{1},\ldots ,\lambda_{6}\right)$. A staged tuning procedure is adopted for reproducibility. The inner-loop weights are initialized by Bryson-rule normalization, with diagonal entries set inversely proportional to the squared operating range of each quantity. $\left(Q_{x},Q_{v},Q_{f}\right)$ are then increased until acceptable tracking is achieved under a nominal assist profile, and $\left(R,\omega_{A}\right)$ are selected such that $A_{k}$ remains small during low-error phases without premature saturation. The outer coefficients are tuned last: $\lambda_{3}$ and $\lambda_{4}$ set the controller’s responsiveness to changes in task-direction user effort, while $\lambda_{5}$ and $\lambda_{6}$ suppress high-frequency oscillations in $A_{k}$ and $B_{k}$. Because abrupt variations in robot assistance directly affect the commanded force, $\omega_{\Delta A}$ is set larger than $\omega_{\Delta B}$.

### 3.3. Energy Tank-Based Passivity Constraints for RMS-AAN

Conventional fixed-parameter admittance control preserves passivity at the interaction port under nominal conditions. In the proposed architecture, however, independent regulation of $A_{k}$ and $B_{k}$ may inject net energy into the environment. To limit such injection while preserving a convex QP structure, the tank constraint is formulated in a conservative sample-and-hold manner using measurements available at the current control step [18,23].

Let $F_{\mathrm{cmd},k}$ denote the actuator-generated command force. The physical dynamics at the current step are written as

(16) $$M_{v}{\ddot{x}}_{a}+B_{v}{\dot{x}}_{a}=F_{\mathrm{cmd},k}+F_{\mathrm{inter},k}$$

Comparing (16) with the two-channel admittance model yields

(17) $$P_{act,k}={\dot{x}}_{a,k}^{⊤}F_{\mathrm{cmd},k}={\dot{x}}_{a,k}^{⊤}\left(A_{k}{\hat{F}}_{\mathrm{robot},k}+\left(B_{k}-1\right)F_{\mathrm{inter},k}\right)$$

for the actuator power.

Let $E_{k}$ denote the energy stored in the tank. Net energy injection extracts energy from the tank, whereas virtual damping recharges it:

(18) $$E_{k+1}=E_{k}+T_{s}{\dot{x}}_{a,k}^{⊤}B_{v}{\dot{x}}_{a,k}-T_{s}{\left[P_{act,k}\right]}_{+}\geq 0$$

Because ${\left[P_{act,k}\right]}_{+}$ is piecewise defined, a non-negative auxiliary variable is introduced:

(19) $$p_{k+i|k}\geq P_{act,k+i|k},\;p_{k+i|k}\geq 0,\;i=0,1,\ldots ,N-1$$

and the conservative tank update is imposed along the horizon as

(20) $$E_{k+i+1|k}=E_{k+i|k}+T_{s}{\dot{x}}_{a,k}^{⊤}B_{v}{\dot{x}}_{a,k}-T_{s}p_{k+i|k}\geq 0,\;i=0,1,\ldots ,N-1$$

Here, the current measured velocity ${\dot{x}}_{a,k}$ is held constant within the recharge term for all horizon steps. This preserves the linearity of the QP and yields a conservative passivity-preserving constraint.

The gain bounds are also enforced over the prediction horizon:

(21) $$0\leq A_{k+i|k}\leq 1,\;0\leq B_{k+i|k}\leq 1,\;i=0,1,\ldots ,N-1$$

The use of the current measured velocity in (20) introduces conservativeness in three ways: $p_{k+i|k}$ upper-bounds the positive actuator power; the damping-based recharge term is computed from the current rather than the predicted velocity, tending to underestimate cumulative dissipation; and tank non-negativity is enforced stepwise over the horizon. Consequently, (19) and (20) provide a conservative sampled-data passivity condition [24], although assistance may be throttled somewhat earlier than under a fully predictive nonlinear tank. The initial energy $E_{0}$ and the upper bound $E_{\max}$ serve as practical stability margins: increasing $E_{0}$ permits longer transient phases of net assistance, while $E_{\max}$ prevents unbounded accumulation during dissipative intervals.

The final optimization problem is formulated as

(22) $$\begin{array}{cccc} \; & \min_{{\left\{u_{k+i|k}\right\}}_{i=0}^{N-1},\;{\left\{p_{k+i|k}\right\}}_{i=0}^{N-1}}\; & \; & \lambda_{1}J_{track}+\lambda_{2}J_{assist}+\lambda_{3}J_{align}+\lambda_{4}J_{A}+\lambda_{5}J_{\Delta A}+\lambda_{6}J_{\Delta B}\\ \; & s.t.\; & \; & \mathrm{prediction}\;\mathrm{model}\;\mathrm{in}\;(4),\\ \; & \; & & \mathrm{tank}\;\mathrm{and}\;\mathrm{gain}\;\mathrm{constraints}\;\mathrm{in}\;(19)–(21). \end{array}$$

### 3.4. MPC Prediction Structure, Input Parameterization, and Stability

The proposed controller follows a standard receding-horizon MPC structure. At each sampling instant *k*, the QP in (22) is solved over a horizon *N*; only the first control move $u_{k|k}^{*}$ is applied, and the optimization is repeated at the next step using the updated state and interaction force. The previous optimal input sequence is shifted by one step and used as both the solver warm start and the nominal input sequence for (9), following standard real-time MPC practice [25,26,27].

The input sequence $u_{k+i|k}={\left[A_{k+i|k},\;B_{k+i|k}\right]}^{⊤}$ is parameterized in dense form, yielding 2N scalar gain variables. Together with the *N* auxiliary tank variables $p_{k+i|k}$, the resulting QP contains 3N scalar decision variables. No basis reduction is imposed because the selected horizon N=20 remains computationally tractable in real time, while dense parameterization preserves the stepwise flexibility of the two gain channels.

Formal closed-loop stability under arbitrary exogenous interaction forces and admissible gain sequences is nontrivial because the effective interaction admittance is modulated directly by the MPC output. Practical stability is instead promoted by three complementary mechanisms: (i) the terminal penalty $Q_{f}$ in (6), which encourages end-of-horizon contraction toward the reference; (ii) the energy-tank constraints in (19) and (20), which limit net energy injection at the interaction port; and (iii) the smoothing penalties $J_{\Delta A}$ and $J_{\Delta B}$, which suppress rapid variation in the effective admittance.

## 4. Results

**Figure 3:**
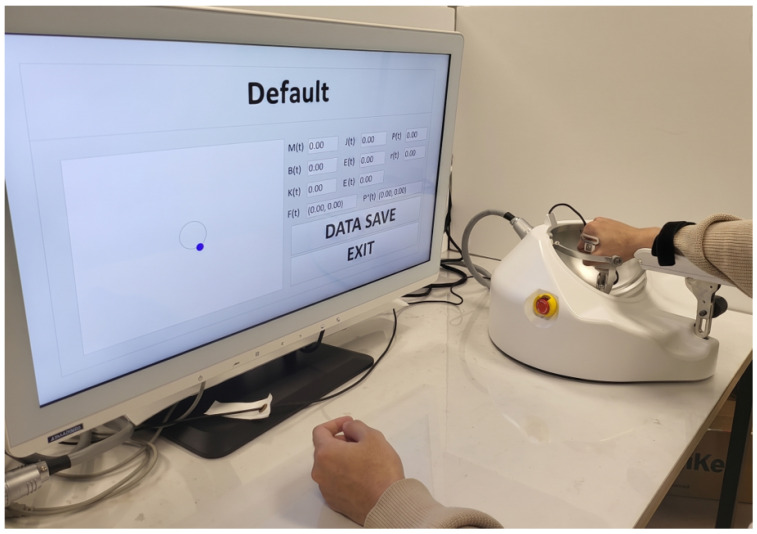
Experimental setup; the planar trajectory-tracking task using the mobile upper-limb rehabilitation robot.

This section evaluates whether the proposed controller realizes AAN behavior while explicitly reflecting reduced user participation. The goal is to verify whether the two-channel structure in Section 3 yields differentiated adaptation of $A_{k}$ and $B_{k}$ under controlled and reproducible conditions. As shown in Figure 3, the experimental platform consisted of a 5-DOF mobile upper-limb rehabilitation robot and a game-based visual interface. The evaluation focused on planar motion in the *x* and *y* directions; the spherical manipulator was excluded from active control, and $F_{\mathrm{inter}}$ was defined as the resultant of the two F/T sensor measurements. The participant was instructed to follow the target point (red) with the current cursor (blue) by generating an appropriate interaction force. The admittance parameters were $M_{v}=2I_{2}$ and $B_{v}=40I_{2}$, and the internal gains were $K_{p}=70I_{2}$ and $K_{d}=10I_{2}$. The MPC parameters and implementation details are listed in Table 1 and Table 2, respectively.

The target trajectory was a circular path with a radius of 0.12m, repeated nine times over 270s. The instructed contribution condition was changed every 90s, resulting in three sequential conditions: insufficient, appropriate, and excessive task-direction contribution.

### 4.1. Experimental Validation of Assist-as-Needed Behavior

**Figure 4:**
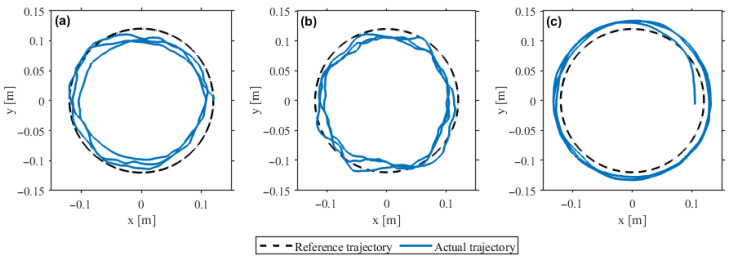
Trajectory-tracking results under three levels of user contribution: (**a**) insufficient, (**b**) appropriate, and (**c**) excessive.

**Figure 5:**
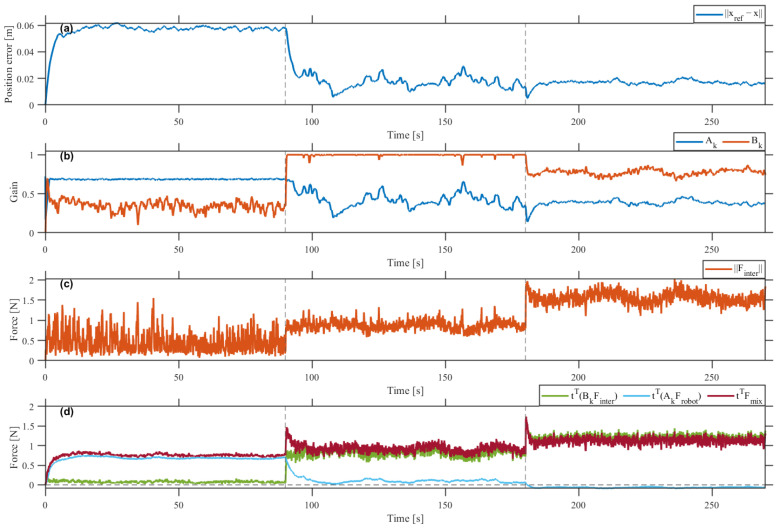
Time-domain responses for experimental validation of Assist-as-Needed behavior: (**a**) position error, (**b**) gain trajectories $A_{k}$ and $B_{k}$, (**c**) interaction-force magnitude $∥F_{\mathrm{inter}}∥$, and (**d**) task-direction force components $t^{⊤}\left(B_{k}F_{\mathrm{inter}}\right)$, $t^{⊤}\left(A_{k}F_{\mathrm{robot}}\right)$, and $t^{⊤}F_{\mathrm{mix}}$.

Figure 4 shows that the actual trajectory followed the reference circular path stably across all three intervals, with the largest deviation occurring under insufficient contribution and the smallest under appropriate contribution. The quantitative results in Table 3 agree with the time histories in Figure 5. The RMS position error decreased monotonically across the three intervals, from 56.59mm under insufficient contribution to 19.23mm under appropriate contribution and 16.81mm under excessive contribution. Tracking accuracy thus improved as user contribution increased, rather than degrading as an error-driven AAN law that trades tracking performance for participation would predict.

The gain trajectories in Figure 5b show an opposite redistribution across the two channels. The mean robot-assist gain $A_{k}$ decreased from 0.682 to 0.426 and 0.382, while the mean participation-reflection gain $B_{k}$ increased from 0.347 to 0.995 and then decreased to 0.775. The non-monotonic behavior of $B_{k}$ in the third interval indicates that, under excessive user task-direction force, the controller regulated the total task-direction force rather than further amplifying $F_{\mathrm{inter}}$ further.

To test whether these internal gain changes reflect an actual shift in user effort, the task-direction participation ratio $\rho_{\mathrm{human}}=t^{⊤}\left(B_{k}F_{\mathrm{inter}}\right)/t^{⊤}F_{\mathrm{mix}}$ was computed from measured forces outside the optimization loop. It evolved as 0.103→0.879→1.000 across the three intervals, closely tracking the internally optimized $B_{k}$. This agreement confirms that the gain trajectories reflect genuine task-direction human contribution rather than an artifact of the cost formulation.

Figure 5c,d corroborate these findings. The interaction-force norm $∥F_{\mathrm{inter}}∥$ grew across intervals, and the dominant task-direction component shifted from $t^{⊤}\left(A_{k}F_{\mathrm{robot}}\right)$ to $t^{⊤}\left(B_{k}F_{\mathrm{inter}}\right)$, becoming predominantly negative in the third interval. The net robot command work $W_{\mathrm{robot}}=\int v^{⊤}F_{\mathrm{cmd}}\;dt$, with $F_{\mathrm{cmd}}=A_{k}F_{\mathrm{robot}}+\left(B_{k}-1\right)F_{\mathrm{inter}}$, decreased from 1.478 to 0.268 and then to −0.150J, confirming a transition from assistive to resistive robot behavior once user contribution exceeded the task requirement. Across all intervals, $t^{⊤}F_{\mathrm{mix}}$ remained bounded, consistent with stable task execution.

### 4.2. Comparative Evaluation Under Induced Motor Slacking

**Figure 6:**
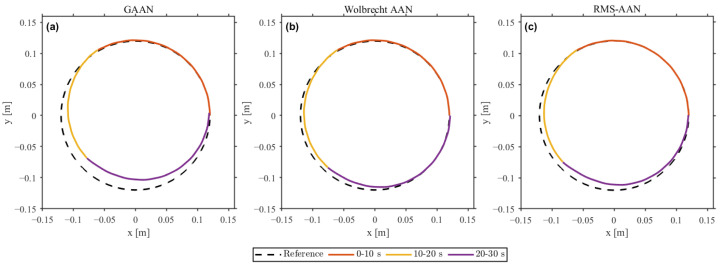
Planar trajectory tracking under induced motor slacking: (**a**) GAAN, (**b**) Wolbrecht-AAN, and (**c**) RMS-AAN. Colors indicate the sub-intervals: 0–10, 10–20, and 20–30 s.

**Figure 7:**
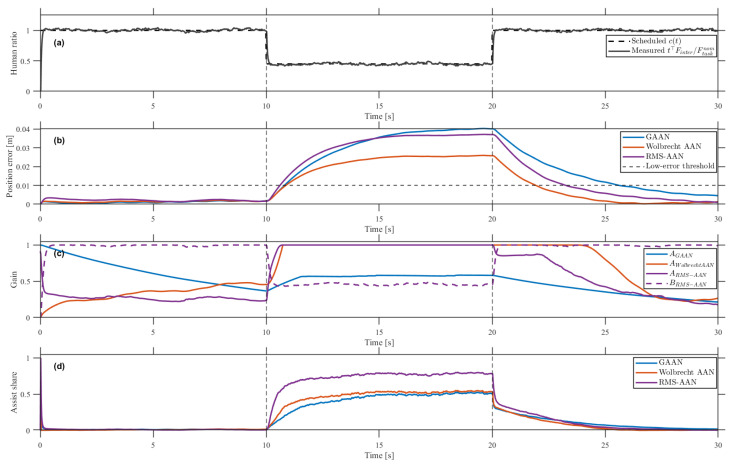
Time-domain comparison of GAAN, Wolbrecht-AAN, and the proposed RMS-AAN under induced motor slacking: (**a**) scheduled contribution c(t) (dashed) vs. measured task-direction contribution ratio (solid); (**b**) position error with the low-error threshold; (**c**) gain trajectories $A_{\mathrm{GAAN}}$, $A_{\mathrm{Wolbrecht}-\mathrm{AAN}}$, $A_{\mathrm{RMS}-\mathrm{AAN}}$, and $B_{\mathrm{RMS}-\mathrm{AAN}}$; (**d**) assist share.

Figure 6 and Figure 7 compare three controllers under induced motor slacking: the error-based AAN (GAAN), the forgetting-factor AAN of Wolbrecht et al., and the proposed RMS-AAN. An identical synthesized interaction-force profile was applied to all three controllers. The task direction t followed the reference-velocity direction, and the task-direction component of $M_{v}a_{\mathrm{ref}}+B_{v}v_{\mathrm{ref}}$ served as the nominal task demand. The principal parallel component was scaled by a contribution schedule c(t): c(t)=1.0 from 0 to 10s and from 20 to 30s, and c(t)=0.45 during the slacking interval from 10 to 20s. Drift, tremor, perpendicular components, noise, delay, and low-pass filtering were superimposed to emulate realistic variations. Since all controllers received the same input, the differences in Figure 7b–d reflect controller structure rather than input variability.

The externally defined references in Figure 7a provide an independent indication of reduced user contribution. The scheduled contribution c(t), shown as the dashed curve, was imposed offline and shared across controllers, whereas the measured task-direction contribution ratio $t^{⊤}F_{\mathrm{inter}}/F_{\mathrm{nom}}$, shown as the solid curve, was computed from the interaction force and reference kinematics without using internal controller variables. From 10 to 20s, both references dropped to approximately 0.45, defining the low-participation interval independently of controller states. These references substitute for direct behavioral or physiological indices such as surface EMG, task-completion latency, or self-reported effort; cross-validation using such external indices is left for future work.

The three controllers differ in how they respond to the imposed contribution reduction. GAAN determines assistance solely from tracking error via $F_{\mathrm{mix}}=F_{\mathrm{inter}}+A_{\mathrm{err}}F_{\mathrm{robot}}$. Wolbrecht-AAN extends this structure with a recursive forgetting mechanism such that assistance decays when the user performs well, although the adjustment remains driven by tracking-error evolution. RMS-AAN uses $F_{\mathrm{mix}}=A_{k}F_{\mathrm{robot}}+B_{k}F_{\mathrm{inter}}$ and jointly optimizes $A_{k}$ and $B_{k}$, explicitly separating assistance from participation reflection.

All three controllers preserved the overall circular trajectory in Figure 6, but the time-domain responses in Figure 7 reveal clear structural differences. From 10 to 20s, the measured contribution dropped to about 0.45. GAAN rapidly drove $A_{\mathrm{GAAN}}$ toward 1, compensating almost immediately and masking the low-participation state behind a small tracking error. Wolbrecht-AAN showed a delayed response: its tracking error increased transiently at slacking onset since the forgetting-based update did not compensate instantaneously, but the assist gain eventually rose to suppress the residual error. RMS-AAN instead redistributed the controller allocation by decreasing $B_{k}$ and increasing $A_{k}$, making the participation reduction explicit rather than concealing it behind a low tracking error.

Table 4 summarizes the descriptive trade-offs. RMS-AAN yielded an RMS tracking error of 31.85mm, comparable to GAAN at 32.46mm and modestly higher than Wolbrecht-AAN at 22.24mm, while producing the lowest aggregate participation ratio $\rho_{\mathrm{human}}=0.282$. This lower value indicates that the externally reduced contribution was reflected more explicitly rather than masked by assistive compensation. The larger $\rho_{\mathrm{human}}$ values of GAAN and Wolbrecht-AAN do not imply superior engagement, since both baselines compensated through error-driven assistance. Under identical synthesized inputs, RMS-AAN thus represented the low-participation state more clearly in the controller allocation, whereas the baselines concealed it through increased assistance. Because the evaluation involved a single participant under a controlled input design, inferential statistics were not performed, and the reported metrics should be interpreted descriptively.

These results should be interpreted as controller-level evidence under controlled conditions. The single-participant setting and the imposed slacking profile were deliberate choices that ensured identical inputs across controllers; thus, the observed differences reflect controller structure rather than inter-subject variability or voluntary behavioral differences. This design, however, cannot reproduce the voluntary adaptation dynamics or the qualitatively different force-production patterns, such as spasticity, abnormal synergies, and proprioceptive deficits, that characterize neurologically impaired users. A multi-participant protocol comprising a healthy pilot cohort followed by a sub-acute stroke cohort, together with the external indices mentioned above, is planned as the next phase of this work.

## 5. Conclusions

This paper proposed an MPC-based AAN control framework for upper-limb rehabilitation robots that explicitly addresses motor slacking. The primary contribution is to operationalize motor slacking as an adaptive reduction in task-direction human contribution under matched task conditions. RMS-AAN decouples the robot-assistance and user-participation channels within a single QP-compatible MPC that jointly handles trajectory tracking, participation alignment, assistance suppression, error-responsive assist shaping, and gain smoothing. Passivity-preserving linear constraints derived from an energy-tank framework promote stable interaction under time-varying gains.

Experimental results show that the method maintains stable task execution while redistributing human and robotic contributions according to user engagement. Under induced motor slacking, RMS-AAN represented reduced human contribution and increased robotic compensation as distinct quantities, whereas the error-based GAAN baseline and the forgetting-factor AAN of Wolbrecht et al. masked reduced participation behind low tracking error. The proposed approach therefore complements tracking-oriented AAN by making user participation an explicit variable in the controller allocation.

The present validation is limited to a single healthy participant and a synthesized slacking profile; the evidence should be interpreted as controller-level proof-of-concept rather than clinical effectiveness. Future work will extend validation in three directions: multi-participant experiments with a healthy pilot cohort followed by a sub-acute stroke cohort; cross-validation of controller-internal variables against external behavioral and physiological indices such as surface EMG, task-completion latency, and self-reported effort; and long-term clinical investigation across more diverse rehabilitation tasks.

## Figures and Tables

**Table 1:** MPC parameters used in the RMS-AAN controller.

Parameter	Value	Parameter	Value
*N*	20	$Q_{x}$	$120I_{2}$
$Q_{v}$	$10I_{2}$	$Q_{f}$	$250I_{2}$
*S*	0.002	R	$0.0015I_{2}$
$\omega_{\Delta A}$	8.0	$\omega_{\Delta B}$	1.0
$\omega_{A}$	1.0	$\lambda_{1}$	5.0
$\lambda_{2}$	10.0	$\lambda_{3}$	50.0
$\lambda_{4}$	30.0	$\lambda_{5}$	50.0
$\lambda_{6}$	30.0	$K_{A,x}$	0.60
$K_{A,\dot{x}}$	0.30	$E_{0}$	0.2
$E_{\max}$	1.0	$e_{ref}^{x}$	0.03
$e_{ref}^{\dot{x}}$	0.06		

**Table 2:** MPC implementation and computational details.

Item	Value
QP solver	OSQP (sparse, operator-splitting)
Sampling period $T_{s}$	10 ms
Prediction horizon *N*	20
Decision variables	2N gain variables +N auxiliary variables =3N=60
Implementation	C++17, real-time Linux
Computing platform	Intel Core i9-9900K, Ubuntu 22.04 (PREEMPT_RT)
Mean/worst-case solve time	3/5 ms

**Table 3:** Metrics from experimental results.

Metric	Interval 1 (Insufficient)	Interval 2 (Appropriate)	Interval 3 (Excessive)
RMS pos err (mm)	56.59	19.23	16.81
Mean $A_{k}$	0.682	0.426	0.382
Mean $B_{k}$	0.347	0.995	0.775
Participation ratio	0.103	0.879	1.000
Robot work (J)	1.478	0.268	−0.150

**Table 4:** Metrics comparison across methods under induced motor slacking.

Metric	GAAN	Wolbrecht-AAN	RMS-AAN
RMS position error (mm)	32.46	22.24	31.85
Participation ratio $\rho_{\mathrm{human}}$	0.595	0.535	0.282

## Data Availability

The data that support the findings of this study are available from the corresponding author upon reasonable request.
